# Subclinical hypothyroidism would not lead to female sexual dysfunction in Chinese women

**DOI:** 10.1186/s12905-017-0465-0

**Published:** 2018-01-25

**Authors:** Han Luo, Wanjun Zhao, Hongliu Yang, Qianqian Han, Li Zeng, Huairong Tang, Jingqiang Zhu

**Affiliations:** 1 Department of Thyroid & Parathyroid surgery, West China Hospital, Sichuan University, West China Hospital, Guoxue Alley 37#, Chengdu, Sichuan People’s Republic of China Postal code: 610041; 2 Department of Nephrology, West China Hospital, Sichuan University, Sichuan Chengdu, People’s Republic of China; 3 Biostatistics Center, West China Hospital, Sichuan University, Sichuan Chengdu, People’s Republic of China; 4 Health Promotion Center, West China Hospital, Sichuan University, West China Hospital, Guoxue Alley 37#, Chengdu, Sichuan People’s Republic of China Postal code: 610041

**Keywords:** Chinese version of the female sexual function index, Beck depression inventory, Female sexual dysfunction, Subclinical hypothyroidism, Thyroid

## Abstract

**Background:**

There is dearth of research about female sexual dysfunction (FSD), especially in China, because of conservative beliefs. Previous studies indicated the relationship between subclinical hypothyroidism and anxiety and depression. However, there is dearth of research regarding the relationship between subclinical hypothyroidism and FSD in Chinses women.

**Method:**

A hospital-based research was conducted. Female sexual function was measured by CVFSFI which includes 19 items. Participants were identified as FSD if CVFSFI ≤ 23.45. Logistics analysis was used to determine risk factor of FSD. All of them finished CVFSFI, Beck Depression Inventory (BDI) self-reporting questionnaires and had thyroid hormone tests. Based on presence and absence of subclinical hypothyroidism, participants were divided into two groups. Risk factors of FSD were identified.

**Result:**

One thousand one hundred nineteen participants with CVFSFI score 25.8 ± 3.9 were enrolled in final analysis. Incidence of subclinical hypothyroidism and FSD in Chinese women was 15.0% and 26.5% respectively. There were no significant difference between subclinical hypothyroidism and control group in FSFI score and prevalence of FSD. Age, Depression (medium risk) was identified as risk factors for nearly all types of FSD, and Income (ranges from 40,000 to 100,000 RMB/year) as protective factor. Subclinical hypothyroidism had no significant relationship with FSD.

**Conclusion:**

Subclinical hypothyroidism is not the risk factor for FSD in urban women of China.

## Background

Since early 1950s, researchers began to investigate sexual dysfunction. And absolutely most of studies focused on erectile dysfunction, premature ejaculation et al. male sexual issue [1, 2]. However, with increasing socioeconomic status in current society, female health is not an ignorable concern nowadays [3, 4]. Until recent decade, researchers began to shift attention to this topic, and the first female “Viagra”-Flibanserin was approved by American FDA in 2016, but there was a dearth of literature about Chinese female sexual dysfunction (FSD).

Lack of, or significantly decreased sexual interest or arousal manifested by at least three of the following characteristics should be identified as FSD: (i) absent or decreased interest in sexual activity; (ii) absent or decreased sexual or erotic thoughts or fantasies; (iii) no or decreased initiation of sexual activity and typically unreceptive to a partner’s attempts to initiate; (iv) absent or decreased sexual excitement or pleasure during sexual activity in almost all or all (approximately 75%–100%) sexual encounters (in identified situational contexts or, if generalized, in all contexts); (v) absent or decreased sexual interest or arousal in response to any internal or external sexual or erotic cues (eg, written, verbal, or visual); or (vi) absent or decreased genital or non-genital sensations during sexual activity in almost all or all (approximately 75%–100%) sexual encounters (in identified situational contexts or, if generalized, in all contexts) [5]. According to different studies, prevalence of FSD varies from 25%, by Kammerer-Doak, to 63%, by Rogers [6]. So it is a common entity besetting women in worldwide. However, the incidence is uncertain in China, especially after the optimal cutoff value was determined by Ma and his team [7] in 2014 basing on the Chinese Han ethnic, the biggest population in China.

FSD is a multifactorial problem [8, 9]. Previous literatures reported psychological factors (irritation, depression and anxiety et al.) and socioeconomic factors (income, education background and living condition) is associated with FSD [10–12]. Meanwhile, endocrinal messengers e.g. prolactin [13], gonadal hormone [14, 15] usually has impact on female sexual function.

Additionally, thyroid disorder--hypothyroidism is believed to associate with FSD [10, 16]. But relationship between subclinical hypothyroidism (ScHt) and FSD is still not clear because of paucity of evidence. Previously, Ergenekon observed that a significantly more women with ScHt of TSH values >10 mU/L had sexual dysfunction [16]. By contrast, Lee et al. demonstrated that ScHt is not a risk factor for FSD in Korean middle-aged women [17].

Due to the controversies in the topic, a preliminary screening study exploring risk factor for FSD with optimal diagnostic value for Chinese was conducted firstly in China.

## Methods

This hospital-based survey was conducted in Health Promotion Center of West China Hospital between June and December of 2015. The health promotion center provided routine check-ups for each member. As described in previous study [18], all of approached women who meet criteria was included in the final analysis.

The diagnosis of Hashimoto thyroiditis referred to Caturegli P’s review [19]. Included subjects were divided into two groups--ScHT and control group, according to thyroid hormone tests. The reference of thyroid hormone and antibody was the same in previous study [18].

Validated by Sun et al. in 2011 [20], Chinese Version of the FSFI (CVFSFI) was adopted to evaluate female sexual function. According to research of Ma et al. in 2014 [7], female sexual dysfunction was defined as a CVFSFI score ≤ 23.45. And 2.7, 3.15, 4.05, 3.8, 3.6 and 3.8 was chosen as optimal cutoff values for low desire/arousal/lubrication/orgasm/satisfaction/sexual pain in the research. In the study, psychological status was evaluated by second version of Beck Depression Inventory (BDI-II), as described in previous study [18].

SPSS version 21 (SPSS Inc., Chicago, IL) was used in the data analysis. Variables were presented as the mean ± standard deviation or median (interquartile range) and compared by t-test or U-test. Chi-square test was used to compare frequency for categorical variables. Risk factor was identified by logistic regression. *P* value less than 0.05 indicates significance.

## Results

During the enrollment period, a total of 1314 women approved to participate in the study. And 1265 women completed the CVFSFI questionnaires in high quality. Then 146 participants were excluded since overt hyper- or hypothyroidism. Finally, 1119 participants came into final analysis, including 168 ScHt participants (15.0%). The baseline were shown in Table 1.

**Table 1:** Basic characters comparison between subclinical hypothyroidism and control group

	ScHT (*N* = 168)	Control (*N* = 951)	*P* value
Age	39.2 ± 7.6	38.5 ± 7.7	0.548
Height	158.2 ± 4.8	158.9 ± 5.2	0.416
Weight	55.3 ± 8.1	54.7 ± 6.6	0.561
BMI	22.1 ± 3.0	21.7 ± 2.4	0.304
fT3	5.0 ± 0.6	5.0 ± 0.7	0.999
fT4	16.4 ± 2.1	17.1 ± 2.2	0.045
Systolic pressure	109.6 ± 15.7	108.6 ± 13.1	0.631
Diastolic pressure	69.9 ± 10.2	69.0 ± 8.9	0.500
TSH	5.4(4.7, 6.5)	2.3(1.7, 2.9)	<0.001
TPOab	9.0(5.2, 41.5)	7.9(5.2,13.1)	0.124
Tgab	19.6(14.9, 149.9)	17.9(13.8,23.5)	0.022
Hypertension (Y/N)	12/156 (7.14%)	47/904 (4.94%)	0.259
Diabetes (Y/N)	6/162 (3.57%)	32/919 (3.36%)	0.819
Hashimoto Thyroiditis (Y/N)	24/144 (14.28%)	50/901 (5.25%)	<0.001
Perimenopause (Y/N)	24/144 (14.28%)	201/750 (21.14%)	0.047
Menopause (Y/N)	6/162 (3.57%)	28/923(2.94%)	0.627
Smoking			0.603
Never	168 (100%)	944 (99.26%)	
Current	0	7	
Alcohol consumption			0.446
Never	162 (96.43%)	900 (94.64%)	
Rarely	6 (3.57%)	51 (5.36%)	
Marital status			0.016
Married	168 (100%)	921 (96.85%)	
Unmarried	0	30 (3.15%)	
Education status			0.401
≤ Middle School	81 (48.21%)	423 (44.48%)	
≥ University	87 (51.79%)	528 (55.52%)	
Income (RMB/year)			0.607
≤ 40,000	30 (17.86%)	207 (21.77%)	
40,000–100,000	132 (78.57%)	693 (72.87%)	
≥ 100,000	6 (3.57%)	51 (5.36%)	
Physical activity			0.552
No	36 (21.43%)	174 (18.30%)	
Rarely	45 (26.79%)	276 (29.02%)	
Frequently	87 (51.79%)	501 (52.68%)	
Depression status			0.727
No	132 (78.57%)	774 (81.39%)	
Light	33 (19.64%)	147 (15.46%)	
Medium	3 (1.79%)	30 (3.15%)	
Severe	0	0	
Parity^a^	1 (1,1)	1 (1,1)	0.162

*ScHT* subclinical hypothyroidism, *fT3* free triiodothyronine, *fT4* free thyroxine, *BMI* body mass index

*P* < 0.05 shows significant difference

^a^Compared by U-test

Significantly more participants with Hashimoto Thyroiditis (HT) in ScHT group than control, 14.28% (24/168) vs 5.25% (50/951) respectively, (*p* < 0.001). Table 1. Besides, significantly more participants were perimenopaused and married in ScHT group, compared with control group, *p* = 0.047, 0.016, respectively.

With respect to FSFI score, a total of 297 participants (26.5%) were at risk for FSD (FSFI score ≤ 23.45), including 36 in ScHT group (21.4%) and 261 in control group (27.4%), no significant difference (*p* = 0.108). Incidence of low desire/ low arousal/ low lubrication/ low orgasm/ low satisfaction and sexual pain in ScHT group was 14.3%, 17.9%, 21.4%, 21.4%, 23.2% and 23.2% respectively. Comparatively, it was 17.7%, 16.0%, 19.6%, 22.1%, 22.7% and 23.7% respectively in control group. Therefore, two groups had no significant difference in FSD and subtype of FSD, as shown in Table 2.

**Table 2:** CVFSFI score and FSD comparison between subclinical hypothyroidism and control group

	ScHT (*N* = 168)	Control (*N* = 951)	*P* value
Total CVFSFI Score	25.8 ± 3.9	25.7 ± 3.9	0.869
Desire	3.5 ± 0.84	3.4 ± 0.8	0.466
Arousal	3.9 ± 1.0	3.9 ± 0.9	0.844
Lubrication	4.9 ± 0.9	4.9 ± 0.9	0.919
Orgasm	4.3 ± 0.9	4.4 ± 0.8	0.628
Satisfaction	4.6 ± 0.9	4.6 ± 0.8	0.780
Pain	4.6 ± 1.0	4.5 ± 0.9	0.622
FSD (CVFSFI total score ≤ 23.45)	36(21.4%)	261(27.4%)	0.108
Low Desire (≤2.7)	24(14.3%)	168(17.7%)	0.319
Low Arousal (≤3.15)	30(17.9%)	152(16.0%)	0.571
Low Lubrication (≤4.05)	36(21.4%)	186(19.6%)	0.600
Low Orgasm (≤3.8)	36(21.4%)	210(22.1%)	0.920
Low Satisfaction (≤3.6)	39(23.2%)	216(22.7%)	0.921
Sexual Pain (≤3.8)	39(23.2%)	225(23.7%)	1.000

*ScHT* subclinical hypothyroidism, *CVFSFI* Chinese Version of Female Sexual Function Index, *FSD* female sexual dysfunction

*P* < 0.05 shows significant difference

Adjusted by cofounding variables, it was clear to find that Age was a common risk factor for FSD. Adjusted odd ratio (OR) was 1.086 (1.021, 1.155), 1.087 (1.023, 1.155), 1.118 (1.050, 1.191) and 1.059 (1.001, 1.120) in Desire, Arousal, Lubrication and FSD, respectively, Table 3. Likely, Depression was common risk factor for all types of FSD. And it is interesting to see that higher Income (40,000–100,000 RMB/year) was a protective variable for nearly all types of FSD. However, it would impair female sexual ability when income >100,000 RMB/year. BMI was identified as risk factor for Low Desire (1.281 [1.121, 1.463], *p* < 0.001). Besides, we failed to found a determined relationship between ScHT with any type of FSD. Table 3.

**Table 3:** Risk factor identification by Enter Multivariate analysis

	Low Desire		Low Arousal		Low Lubrication		Low Orgasm		Low Satisfaction		Sexual Pain		FSD	
Variables	OR (95% CI)	*P* value	OR (95% CI)	*P* value	OR (95% CI)	*P* value	OR (95% CI)	*P* value	OR (95% CI)	*P* value	OR (95% CI)	*P* value	OR (95% CI)	*P* value
Age	1.086 (1.021, 1.155)	0.008	1.087 (1.023, 1.155)	0.007	1.118 (1.050, 1.191)	0.001	1.019 (0.964, 1.078)	0.500	1.039 (0.981, 1.101)	0.188	0.994 (0.935, 1.056)	0.837	1.059 (1.001, 1.120)	0.045
Income (40,000–100,000 RMB/year)	0.443 (0.212, 0.927)	0.031	0.397 (0.195, 0.810)	0.011	0.298 (0.148, 0.597)	0.001	0.551 (0.289, 1.049)	0.070	0.328 (0.174, 0.620)	0.001	0.576 (0.305, 1.090)	0.090	0.308 (0.164, 0.579)	<0.001
ScHT	0.874 (0.272, 2.807)	0.821	1.045 (0.34, 3.209)	0.939	1.716 (0.575, 5.121)	0.333	0.700 (0.247, 1.979)	0.501	1.064 (0.387, 2.925)	0.904	0.412 (0.128, 1.325)	0.137	0.529 (0.175, 1.603)	0.260
HT	0.442 (0.088, 2.216)	0.321	1.122 (0.319, 3.940)	0.858	1.099 (0.286, 4.223)	0.891	1.377 (0.424, 4.473)	0.594	0.453 (0.092, 2.227)	0.330	0.290 (0.061, 1.381)	0.120	0.681 (0.200, 2.312)	0.537
fT4	0.989 (0.851, 1.151)	0.890	0.906 (0.775, 1.058)	0.213	1.089 (0.941, 1.260)	0.253	0.970 (0.850, 1.107)	0.653	0.863 (0.750, 0.993)	0.040	0.946 (0.830, 1.079)	0.411	0.94 (0.824, 1.072)	0.357
Tgab	1.001 (1.000, 1.002)	0.078	1.001 (1.000, 1.002)	0.168	1.000 (0.997, 1.002)	0.691	1.001 (1.000, 1.002)	0.062	0.999 (0.997, 1.001)	0.413	1.000 (0.999, 1.001)	0.668	1.000 (1.000, 1.001)	0.378
TSH	0.944 (0.795, 1.122)	0.516	0.931 (0.769, 1.127)	0.461	0.953 (0.789, 1.151)	0.618	1.097 (0.927, 1.298)	0.281	0.965 (0.816, 1.140)	0.671	1.202 (0.986, 1.465)	0.068	1.031 (0.863, 1.231)	0.739
Depression status (medium risk)	2.260 (0.5, 10.205)	0.289	8.048 (1.853, 34.950)	0.005	20.601 (3.649, 116.316)	0.001	3.402 (0.882, 13.119)	0.075	4.948 (1.188, 20.609)	0.028	1.716 (0.42, 7.012)	0.452	33.035 (3.553, 307.108)	0.002
Education background (≥University)	1.534 (0.72, 3.267)	0.268	0.610 (0.289, 1.286)	0.194	1.233 (0.593, 2.563)	0.574	0.916 (0.471, 1.779)	0.795	0.848 (0.431, 1.668)	0.634	0.783 (0.404, 1.517)	0.468	0.783 (0.404, 1.517)	0.468
Perimenopause	0.875 (0.344, 2.231)	0.780	0.546 (0.217, 1.376)	0.200	0.754 (0.303, 1.877)	0.545	1.264 (0.532, 3.006)	0.596	2.618 (1.061, 6.458)	0.037	1.173 (0.507, 2.718)	0.709	1.173 (0.507, 2.718)	0.709
BMI	1.281 (1.121, 1.463)	<0.001	1.086 (0.933, 1.223)	0.339	1.103 (0.964, 1.262)	0.155	1.075 (0.958, 1.207)	0.217	0.954 (0.847, 1.076)	0.446	1.067 (0.947, 1.203)	0.285	1.067 (0.947, 1.203)	0.285
Smoking	2.325 (0.241, 22.401)	0.465	1.224 (0.076, 19.821)	0.887	1.014 (0.059, 17.577)	0.992	3.103 (0.463, 20.787)	0.243	4.057 (0.615, 26.775)	0.146	3.024 (0.392, 23.342)	0.289	3.024 (0.392, 23.342)	0.289

*ScHT* subclinical hypothyroidism, *HT* Hashimoto Thyroiditis, *fT4* free thyroxine, *OR* odd ratio, *CI* confidence interval, *FSD* female sexual dysfunction

*P* < 0.05 shows significant difference

## Discussion

To our limited knowledge, it is the first study to explore the relationship between thyroid hormone and female sexual dysfunction (FSD) in China. Though Lee et al. firstly has proved subclinical hypothyroidism (ScHT) is not risk factor for FSD [17], they failed to set the relationship between thyroid hormone and FSD. At the same time, our study also is the first screening research based on the Ma et al.’s optimal cutoff value after 2014 [7, 20].

In our current study, it is interesting to know that the incidence of subclinical hypothyroidism and female sexual dysfunction is 15.0% and 26.5% respectively. As we know, the prevalence of subclinical hypothyroidism is higher than previous reports [21, 22], which is mainly resulted from iodine deficiency. Sichuan province locates at western inside mountainous land of China, and element iodine is relative deficient, so government forces to sell iodized salt for 16 years. Incidence of iodine deficiency-related thyroid disease decreases much [23]. Meanwhile, there was no significant difference between ScHT group and control group in Female Sexual Function Index (FSFI) score (25.8 ± 3.9 vs 25.7 ± 3.9). ScHT was not a risk factor for FSD even though after adjustment, which confirmed the Lee’s findings [17].

Our study suggested that less than one third—26.5% of women was beset by FSD. Reportedly, Laumann et al. found that 43% of American women was at risk for FSD [24]. 52% of interviewed women had at least one type of FSD in an Iranian population-based study [25]. The number in Egypt is 52.8% according to Ibrahim et al. report [26]. In all, the difference was mainly resulted from different ethnics, cutoff value and conservative culture background---Chinese women are reluctant to talk it with physicians. On the other hand, it coincided with researches in Iran, Turkey, Medellin and Malaysia, which reported the prevalence was 31.5%, 26.1%, 30%, 29.6% respectively [27–30]. Though previous research conducted by Sefa Resim et al. found that education level of male and female was found to be significantly related to FSD [31], we found higher education (≥University) was not protective factor for FSD. This may be resulted from different classifier—in Sefa Resim’s report, participants was divided into illiterate, schooling < 8 years and >8 years.

Subclinical hypothyroidism, affecting 10% of population, is more frequently detected in women than men, and advanced age [21, 32]. Due to the influence of thyroid hormone on lipid metabolism, subclinical hypothyroidism is proved to be risk factor for heart failure, coronary heart disease and dyslipidemia [33–37]. But the research about the relationship between subclinical hypothyroidism and FSD is rare. In previous study, Atis G et al. reported that a significant percent of women with clinic hypothyroidism and subclinical hypothyroidism with TSH values > 10 mIU/L had sexual dysfunction. Subclinical hypothyroidism, however, with TSH values < 10 mIU/L did show this trend [16]. It shared the same view with our study, because only 6 participant with TSH more than10 mIU/L were enrolled in our study. Thus TSH in absolutely most of participants was lower than 10 mIU/L in the present study. And it is found that ScHT is not risk factor for any type of FSD. Therefore, the result, in fact, kept consistent with Atis G’s.

Several limitations are inevitable in our study. First of all, it is limited by the location. West China Hospital locates southwestern part of China. People intend to be more conservative than in Beijing or costal region, especially to sexual life or experience, because of comparatively less developed economy. Reportedly, Sun et al. found prevalence of FSD was 52% in Beijing, but they used DSM-IV-TR, not FSFI as screening tool. And the result in our study hardly has general representativeness for a whole population due to uneven development of economy and culture. Therefore, we look forward more researchers could focus on this topic. Secondly, short of diversity is inevitable, because the absolutely most of participants come from urban region. The result may be a little different when adopted in urban and suburban area. Therefore, a multicenter screening study is needed. Thirdly, due to the limitation of health examination programs, sex hormone, like testosterone, estradiol, luteinizing hormone et al., cannot be collected. An extensive study involving sex hormone is conducting in department.

## Conclusions

In conclusion, the prevalence of female sexual dysfunction and subclinical hypothyroidism in China, southwestern part of China exactly, was 26.5% and 15.0%, respectively. And subclinical hypothyroidism is not risk factor for any type of FSD.

## Abbreviations

BDI: Beck Depression Inventory
CVFSFI: Chinese version of the female sexual function index
FSD: Female sexual dysfunction.
